# Demographic and Clinicopathologic Factors Associated With Colorectal Adenoma Recurrence

**DOI:** 10.1001/jamanetworkopen.2025.56853

**Published:** 2026-02-04

**Authors:** Usman Ayub Awan, Qingyuan Song, Kristen K. Ciombor, Adetunji T. Toriola, Jungyoon Choi, Timothy Su, Xiao-ou Shu, Kamran Idrees, Kay M. Washington, Wei Zheng, Wanqing Wen, Zhijun Yin, Xingyi Guo

**Affiliations:** 1 Division of Epidemiology, Department of Medicine, Vanderbilt-Ingram Cancer Center, Vanderbilt University Medical Center, Nashville, Tennessee; 2 Department of Medical Laboratory Technology, The University of Haripur, Haripur, Khyber Pakhtunkhwa, Pakistan; 3 Department of Biomedical Informatics, Vanderbilt University Medical Center, Nashville, Tennessee; 4 Department of Department of Computer Science, Vanderbilt University, Nashville, Tennessee; 5 Division of Hematology/Oncology, Department of Medicine, Vanderbilt University Medical Center, Vanderbilt-Ingram Cancer Center, Nashville, Tennessee; 6 Division of Public Health Sciences, Department of Surgery, and Siteman Cancer Center, Washington University School of Medicine St Louis, St Louis, Missouri; 7 Division of Oncology and Hematology, Department of Internal Medicine, Korea University Ansan Hospital, Korea University College of Medicine, Ansan, Korea; 8 Division of Surgical Oncology and Endocrine Surgery, Department of Surgery, Vanderbilt University Medical Center, Vanderbilt-Ingram Cancer Center Nashville, Tennessee; 9 Department of Pathology, Vanderbilt University Medical Center, Nashville, Tennessee; 10 Department of Electrical and Computer Engineering, Vanderbilt University, Nashville, Tennessee

## Abstract

**Importance:**

Current colorectal surveillance guidelines emphasize adenoma characteristics but overlook temporal, racial, and sex-based heterogeneity in recurrence risk, a gap that limits equitable and personalized care.

**Objective:**

To evaluate the associations of demographic factors, obesity, and adenoma features with recurrence risk over time in a large longitudinal surveillance cohort.

**Design, Setting, and Participants:**

This retrospective cohort study included adults who underwent their first colonoscopic polypectomy between January 1990 and July 2024 at a tertiary medical center.

**Exposures:**

Demographic variables included race and ethnicity, sex, obesity (body mass index >30), family history of colorectal cancer (CRC) or polyps, and age at adenoma onset (<50 vs ≥50 years). Adenoma features included histology, size, number, and dysplasia.

**Main Outcomes and Measures:**

The primary outcome was recurrence-free survival, defined as time from initial polypectomy to histologically confirmed recurrence. Time-varying coefficient Cox models were fitted to handle the nonconstant associations of exposure over the follow-up time. The follow-up time was categorized into 3 periods (less than 5 years, 5 to 10 years, and 10 or more years). The heterogeneity of exposure associations across the 3 follow-up periods was assessed with likelihood ratio tests.

**Results:**

Among 59 667 patients (mean [SD] age, 60 years [11.2]; 29 401 [49.3%] female; 1007 [1.7%] Asian and Pacific Islander, 646 [1.1%] Hispanic, 5972 [10.0%] non-Hispanic Black, and 52 042 [87.2%] non-Hispanic White; median [IQR] follow-up, 4 [1-9] years), 17 596 (29.5%) experienced overall recurrence within 5 years. High-grade dysplasia demonstrated the largest early phase association (adjusted hazard ratio [aHR], 4.00; 95% CI, 3.56-4.50) with complete midterm and late attenuation, while villous histology exhibited biphasic patterns with early elevation (aHR, 2.89; 95% CI, 2.63-3.18) and late-phase (>10 years) reemergence (aHR, 2.71; 95% CI, 2.15-3.41). Obesity conferred persistent risk across all surveillance intervals (early: aHR, 1.16; 95% CI, 1.11-1.21; late: aHR, 1.22; 95% CI, 1.09-1.35). Female patients with high-risk adenomas exhibited marked late-term (>10 years) elevation exceeding male patients (female patients: aHR, 1.73; 95% CI, 1.43-2.08 vs male patients: aHR, 1.29; 95% CI, 1.06-1.58).

**Conclusions and Relevance:**

Both histopathologic features and demographic factors demonstrated distinct time-dependent patterns in adenoma recurrence, underscoring the need for surveillance strategies that account for temporal variation and population-specific risk profiles.

## Key Points

**Question:**

Which demographic and histopathologic factors are associated with colorectal adenoma recurrence in postpolypectomy surveillance, and how do their outcomes vary over long-term follow-up?

**Findings:**

In this cohort study of 59 667 patients (overall recurrence, 29.5%), high-grade dysplasia showed the strongest association with early recurrence, while villous histology demonstrated a late-phase (>10 years) resurgence. Sex displayed temporal heterogeneity with female patients with high-risk adenomas experiencing higher late-term (>10 years) recurrence risk than male patients.

**Meaning:**

These findings suggest that both histopathologic and demographic factors show time-dependent associations with recurrence, supporting dynamic and individualized surveillance strategies.

## Teaser

This cohort study evaluates the associations of demographic factors, obesity, and adenoma features with colorectal adenoma recurrence risk over time in a large longitudinal surveillance cohort in the US.

## Introduction

Colorectal cancer (CRC) persists as a leading cause of cancer-related mortality globally, with adenomatous polyps representing the primary precursor lesions for 60% to 90% of cases.^1,2^ Current postpolypectomy surveillance guidelines, such as those from the US Multi-Society Task Force (USMSTF) and the European Society of Gastrointestinal Endoscopy (ESGE), stratify recurrence risk primarily based on polyp characteristics, including size, histology, and multiplicity.^1,2,3^ Recent guideline updates reveal persistent inconsistencies: the 2020 ESGE omits surveillance for 1 to 4 nonadvanced adenomas (NAAs), recommending return to routine screening, whereas USMSTF advocates 3- to 10-year surveillance intervals depending on adenoma characteristics.^1,3,4^

These discrepancies underscore a broader limitation: guidelines universally neglect demographic variables (race, sex, family history, age, and obesity), potentially perpetuating inequities.^5,6,7^ Identifying demographic-specific patterns is essential, as uniform polyp-centric guidelines may inadequately address population heterogeneity in recurrence risk and timing. Of note, racial disparities in CRC outcomes are well-documented, with non-Hispanic Black individuals experiencing higher CRC incidence and mortality compared with White populations; however, limited studies have explored the association between demographic disparities and adenoma recurrence.^8,9^ Despite differential recurrence patterns across ethnic groups, current surveillance protocols lack race-specific risk stratification, while central obesity (waist-to-hip ratio) independently increases CRC risk by 15% to 18% per standard deviation, surpassing the effecte of body mass index (BMI).^6,7^

Histopathological features (high-grade dysplasia, villous architecture) have significant effect sizes in adenoma recurrence,^2,9,10^ but their prioritization varies across guidelines. USMSTF classifies villous histology or high-grade dysplasia as high-risk for 3-year surveillance, while ESGE prioritizes size and dysplasia, potentially underestimating villous histology’s long-term impact.^9,10^ Long-term surveillance data from diverse populations remain sparse, restricting insights into how demographic and histopathological factors interact over time.^9,11,12^ Investigating adenoma recurrence demands longitudinal follow-up with serial colonoscopies.

Current literature provides limited insight into temporal recurrence dynamics, constraining long-term surveillance optimization.^1,2,13,14^ While guidelines assume constant risk, it remains unclear whether demographic factors and histopathologic features exert a stable influence or vary across surveillance intervals.^15,16,17^

This study analyzed Vanderbilt electronic health records spanning 3.5 million patients, using individuals undergoing polypectomy to clarify surveillance associations. We employed interval-specific survival analyses (<5, 5-10, >10 years) to evaluate time-varying associations of demographic factors (race and ethnicity, sex, obesity, age, and family history) alongside polyp characteristics as coprimary associations with adenoma recurrence, addressing guideline gaps in equity and personalization.

## Methods

### Study Design and Setting

This study was approved by the Vanderbilt University Medical Center (VUMC) institutional review board with waiver of informed consent due to retrospective design and large sample size. The study followed Strengthening the Reporting of Observational Studies in Epidemiology (STROBE) reporting guidelines. The Vanderbilt Polypectomy Cohort uses clinical data from January 1990 through July 2024, with harmonized extraction protocols ensuring consistency across coding system transitions (*International Classification of Diseases, Ninth Revision [ICD-9]* to *International Classification of Diseases, Tenth Revision [ICD-10]*).^18^

### Data Source

Data were extracted from the VUMC electronic health record (EHR) system (eStar [Epic Systems Corp]), encompassing colonoscopy reports, pathology findings, demographics, and comorbidities. To ensure robustness, a multistep validation process was implemented. First, polyp cases and procedures were identified using standardized diagnostic and procedural codes (*ICD-9*, *ICD-10*, *Current Procedural Terminology*, and Healthcare Common Procedure Coding System) to address discrepancies across coding systems. Second, all polyp diagnoses were cross-verified against original histopathology reports to confirm neoplastic status, histology, and dysplasia grade. Third, an institutional large language model (LLM) extracted adenoma features from unstructured text: histology subtype, dysplasia grade, lesion size, and multiplicity, which were mapped to predefined analytic categories. Validation in 100 patients showed above 95% concordance for histology, multiplicity, and size, with discordant cases adjudicated before dataset finalization.^19^

### Vanderbilt Polypectomy Cohort Identification

The study population included adults aged 18 years or older with histopathologically confirmed colorectal adenoma who underwent polypectomy at VUMC. Exclusion criteria included prior CRC or other cancers, nonneoplastic polyps, colectomy, incomplete colonoscopies, and race and ethnicity classified as other. Race and ethnicity were ascertained from patient self-report at clinic registration via electronic health records and categorized as Asian or Pacific Islander, Hispanic, non-Hispanic Black, non-Hispanic White, or other. The other category, including patients identifying as multiracial, Native American and Alaska Native, Native Hawaiian, or who declined to specify, was excluded due to category heterogeneity.

Colorectal adenomas were categorized as high risk if they met 1 or more criteria: 3 or more in number, size 10 mm or greater, villous or tubulovillous histology, or high-grade dysplasia. Low-risk adenomas were defined as less than 3 adenomas, less than 10 mm in size, and low-grade dysplasia. A multidisciplinary team (U.A.A., Q.S., Z.Y., and X.G.) independently reviewed cohort eligibility to ensure adherence to clinical and histopathologic standards. Additional details on data extraction and variable definitions are provided in the eMethods in Supplement 1.

### Follow-Up and Censoring

Patients were followed from index polypectomy to the earliest of adenoma recurrence, death, loss to follow-up, or study end (July 2024); deaths before a documented recurrence were censored at the date of death. Patients undergoing colectomy or lost to follow-up were censored at their last known clinical encounter. The primary outcome, recurrence-free survival, was defined as the time from initial polypectomy to histologically confirmed recurrence on subsequent colonoscopy (≥6 months postprocedure). This was analyzed both as a binary end point and as time-to-event data to estimate recurrence-free survival.

### Statistical Analysis

Baseline characteristics were summarized using descriptive statistics with categorical variables reported as numbers and percentages. The associations between adenoma recurrence and demographic and clinical factors were estimated using Cox proportional hazards models with adjustment for age at first polypectomy, calendar year of first polypectomy (categorized as <2005, 2005-2009, 2010-2014, 2015-2019, or 2020-2024), sex, and race and ethnicity. The Cox models for adenoma characteristic models were additionally adjusted for obesity, family history of CRC, family history of polyps, nonsteroidal anti-inflammatory drugs (NSAIDs), and aspirin use. Aspirin and NSAID use were ascertained from medication records and problem lists documented after the index polypectomy. The proportional hazards assumption was verified using Schoenfeld residuals. For covariates that did not satisfy the proportional hazards assumption, we fitted stratified Cox models through the use of the strata function (with the R survival package). For the exposure variables of interest, we fitted time-varying coefficient models^20^ to investigate the nonconstant associations of exposure over the follow-up time. The follow-up time was categorized into 3 periods (ie, <5 years, 5-10 years, and >10 years). The heterogeneity of exposure associations across the 3 follow-up periods was assessed with likelihood ratio tests by comparing Cox models with and without interaction terms. We further analyzed the adenoma characteristics and investigated their association heterogeneity across different ethnicities or sexes by stratified analyses and also likelihood ratio tests. Given the large sample size, interpretation of findings prioritized the magnitude of effect sizes (hazard ratios) and clinical relevance over statistical significance alone. Analyses were completed in R version 4.4.3 (R Project for Statistical Computing), and a *P* value less than .05 was considered significant.

## Results

The Vanderbilt polypectomy cohort included 59 667 patients (median [IQR] follow-up, 4 [1-9] years; 6262 deaths [10.5%]) including 30 266 male patients (50.7%), with a racial and ethnic distribution of 1007 (1.7%) Asian or Pacific Islander, 646 (1.1%) Hispanic, 5972 (10.0%) non-Hispanic Black, and 52 042 (87.2%) non-Hispanic White patients (Figure). Early-onset adenoma (<50 years) occurred in 11 018 patients (18.5%) overall, with marked racial variation (Asian and Pacific Islander: 204 patients [20.3%], Hispanic: 197 patients [30.5%], non-Hispanic Black: 1161 patients [19.4%], and non-Hispanic White: 9456 patients [18.2%]). Obesity prevalence demonstrated profound racial disparities (Asian and Pacific Islander: 124 patients [12.3%], Hispanic: 212 patients [32.8%], non-Hispanic Black: 2810 patients [47.1%], and non-Hispanic White: 16 495 patients [31.7%]). Family history of CRC was more frequent in non-Hispanic White (7307 patients [14.0%]) vs other racial and ethnic groups (Asian and Pacific Islander: 104 patients [10.3%], Hispanic: 70 patients [10.8%], and non-Hispanic Black: 752 patients [12.6%]), with similar patterns for polyp history (Asian and Pacific Islander: 10 patients [1.0%], Hispanic: 9 patients [1.4%], non-Hispanic Black: 96 patients [1.6%], and non-Hispanic White: 1472 patients [2.8%]). Both familial factors were more prevalent in female patients (CRC: 4629 female patients [15.7%] vs 3604 male patients [11.9%]; polyps: 943 female patients [3.2%] vs 644 male patients [2.1%]). Adenoma histology varied by sex (tubulovillous: 1293 male patients [4.3%] vs 1033 female patients [3.6%]; mixed: 8450 male patients [27.9%] vs 8005 female patients [27.2%]; serrated: 1730 female patients [5.9%] vs 1014 male patients [3.4%]; and villous: 1033 male patients [3.5%] vs 670 female patients [2.2%]; *P* < .001) and race and ethnicity (Asian and Pacific Islander: highest tubular at 403 patients [40.0%]; non-Hispanic Black: highest tubulovillous at 325 patients [5.4%]; and non-Hispanic White: highest mixed at 177 patients [27.7%] and highest serrated at 2485 patients [4.8%]). High-grade dysplasia was more prevalent in Hispanic (16 patients [2.5%]) and non-Hispanic Black (138 patients [2.3%]) patients vs Asian and Pacific Islander (19 patients [1.9%]) and non-Hispanic White (944 patients [1.8%]) patients. High-risk adenomas were more prevalent in male patients (7645 male patients [25.3%] vs 7009 female patients [23.8%]) (Table 1).

**Figure.**
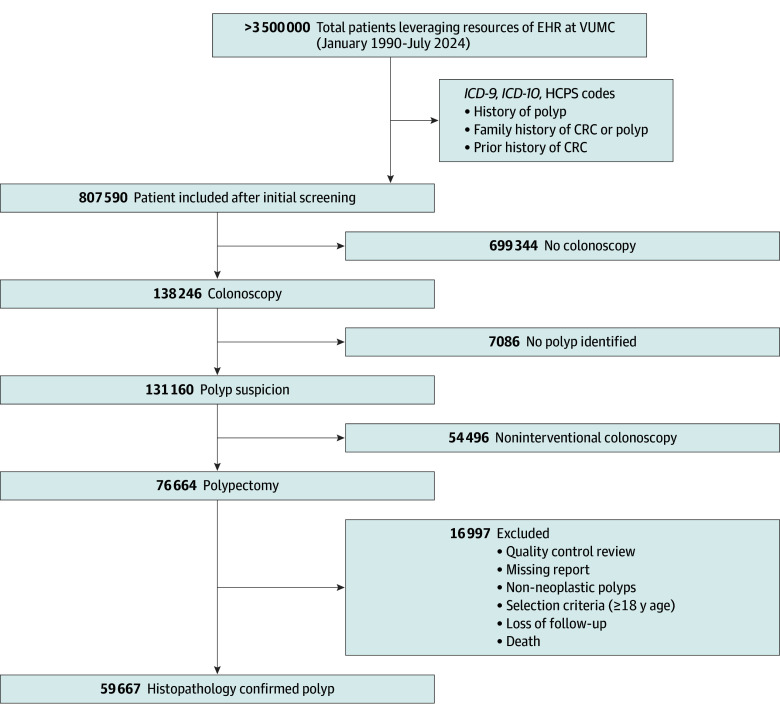
Patient Selection and Cohort Characteristics for the Vanderbilt Polypectomy Cohort EHR indicates electronic health record; CRC, colorectal cancer; HCPCS, Healthcare Common Procedure Coding System;* ICD-9*, *International Classification of Diseases, Ninth Revision*; *ICD-10*, *International Classification of Diseases, Tenth Revision*; VUMC, Vanderbilt University Medical Center.

**Table 1.** Baseline Characteristics of the Study Population Stratified by Race, Ethnicity, and Sex

Variable	Participants, No. (%)					*P* value^a^	Participants, No. (%)		*P* value^a^
	Total No. (N = 59 667)	Ethnicity					Sex		
		Asian or Pacific Islander (n = 1007)	Hispanic (n = 646)	Non-Hispanic Black (n = 5972)	Non-Hispanic White (n = 52 042)		Female (n = 29 401)	Male (n = 30 266)	
Demographics characteristics									
Sex									
Female	29 401 (49.3)	495 (49.2)	351 (54.3)	3435 (57.5)	25 120 (48.3)	<.001	NA	NA	NA
Male	30 266 (50.7)	512 (50.8)	295 (45.7)	2537 (42.5)	26 922 (51.7)		NA	NA	NA
Obesity	19 641 (32.9)	124 (12.3)	212 (32.8)	2810 (47.1)	16 495 (31.7)	<.001	10 423 (35.5)	9218 (30.5)	<.001
Adenoma onset age, y									
0-49	11 018 (18.5)	204 (20.3)	197 (30.5)	1161 (19.4)	9456 (18.2)	<.001	5879 (20.0)	5139 (17.0)	<.001
≥50	48 649 (81.5)	803 (79.7)	449 (69.5)	4811 (80.6)	42 586 (81.8)		23 522 (80.0)	25 127 (83.0)	
Family history of polyps	1587 (2.7)	10 (1.0)	9 (1.4)	96 (1.6)	1472 (2.8)	<.001	943 (3.2)	644 (2.1)	<.001
Family history of colorectal cancer	8233 (13.8)	104 (10.3)	70 (10.8)	752 (12.6)	7307 (14.0)	<.001	4629 (15.7)	3604 (11.9)	<.001
Adenoma characteristics									
Subtypes									
Mixed	16 455 (27.6)	255 (25.3)	156 (24.1)	1644 (27.5)	14 400 (27.7)	<.001	8005 (27.2)	8450 (27.9)	<.001
Not defined	18 750 (31.4)	260 (25.8)	177 (27.4)	1495 (25.0)	16 818 (32.3)		9018 (30.7)	9732 (32.2)	
Serrated	2744 (4.6)	42 (4.2)	26 (4.0)	191 (3.2)	2485 (4.8)		1730 (5.9)	1014 (3.4)	
Tubular	17 666 (29.6)	403 (40.0)	251 (38.9)	2168 (36.3)	14 844 (28.5)		8559 (29.1)	9107 (30.1)	
Tubulovillous	2349 (3.9)	28 (2.8)	24 (3.7)	325 (5.4)	1972 (3.8)		1056 (3.6)	1293 (4.3)	
Villous	1703 (2.9)	19 (1.9)	12 (1.9)	149 (2.5)	1523 (2.9)		1033 (3.5)	670 (2.2)	
Dysplasia									
High grade	1117 (1.9)	19 (1.9)	16 (2.5)	138 (2.3)	944 (1.8)	.04	498 (1.7)	619 (2.0)	.002
Low grade	58 550 (98.1)	988 (98.1)	630 (97.5)	5834 (97.7)	51 098 (98.2)		28 903 (98.3)	29 647 (98.0)	
No. of adenomas									
<3	36 047 (60.4)	718 (71.3)	424 (65.6)	4245 (71.1)	30 660 (58.9)	<.001	18 442 (62.7)	17 605 (58.2)	<.001
≥3	7417 (12.4)	113 (11.2)	85 (13.2)	743 (12.4)	6476 (12.4)		3321 (11.3)	4096 (13.5)	
Not defined	16 203 (27.2)	176 (17.5)	137 (21.2)	984 (16.5)	14 906 (28.6)		7638 (26.0)	8565 (28.3)	
Size of adenoma, mm									
<10	24 670 (41.3)	490 (48.7)	293 (45.4)	2820 (47.2)	21 067 (40.5)	<.001	12 269 (41.7)	12 401 (41.0)	<.001
≥10	6669 (11.2)	104 (10.3)	75 (11.6)	713 (11.9)	5777 (11.1)		3101 (10.5)	3568 (11.8)	
Not defined	28 328 (47.5)	413 (41.0)	278 (43.0)	2439 (40.8)	25 198 (48.4)		14 031 (47.7)	14 297 (47.2)	
Adenoma type									
High risk	14 654 (24.6)	224 (22.2)	167 (25.9)	1542 (25.8)	12 721 (24.4)	.03	7009 (23.8)	7645 (25.3)	<.001
Low risk	45 013 (75.4)	783 (77.8)	479 (74.1)	4430 (74.2)	39 321 (75.6)		22 392 (76.2)	22 621 (74.7)	
Recurrence status									
No recurrence	42 071 (70.5)	747 (74.2)	535 (82.8)	4381 (73.4)	36 408 (70.0)	<.001	21 143 (71.9)	20 928 (69.1)	<.001
Recurrence	17 596 (29.5)	260 (25.8)	111 (17.2)	1591 (26.6)	15 634 (30.0)		8258 (28.1)	9338 (30.9)	

Abbreviation: NA, not applicable.

^a^ Pearson χ^2^ test was used to calculate *P* values for comparisons of categorical variables between groups.

### Associations With Demographic and Clinical Factors

As shown in Table 2, while a relatively constant association between adenoma recurrence and male sex (<5 years: adjusted hazard ratio [aHR], 1.11; 95% CI, 1.07-1.16; 5-10 years: aHR, 1.17; 95% CI, 1.12-1.23) or obesity (5-10 years: aHR, 1.10; 95% CI, 1.05-1.16; >10 years: aHR, 1.22; 95% CI, 1.09-1.35) was observed across the 3 follow-up periods, association of adenoma recurrence with other demographic and clinical factors showed significant heterogeneity across the follow-up periods. Non-Hispanic Black patients exhibited sustained lower recurrence during early and midterm surveillance (<5 years: aHR, 0.89; 95% CI, 0.83-0.96; 5-10 years: aHR, 0.84; 95% CI, 0.77-0.92; both *P* < .001), with nonsignificant late attenuation (aHR, 1.15; 95% CI, 0.98-1.34; *P* = .09). Asian and Pacific Islander patients showed early protection (aHR, 0.80; 95% CI, 0.67-0.96; *P* = .01) with subsequent normalization. Early-onset adenomas showed elevated early (aHR, 1.14; 95% CI, 1.07-1.22; *P* < .001), reduced midterm (aHR, 0.79; 95% CI, 0.73-0.86; *P* < .001), and nonsignificant late risk. Family history of CRC was associated with persistent risk with stronger midterm associations (aHR, 1.44; 95% CI, 1.35-1.53; *P* < .001), while polyp history showed minimal early (aHR, 1.15; 95% CI, 1.03-1.29; *P* = .01) but marked midterm (aHR, 1.45; 95% CI, 1.28-1.65; *P* < .001) elevation.

**Table 2.** Adjusted Hazard Ratios (HR) for Adenoma Recurrence by Demographic and Clinical Variables Across Time Periods^a^

Variables	<5 y		5-10 y		>10 y		Heterogeneity *P* value
	HR (95% CI)	*P* value	HR (95% CI)	*P* value	HR (95% CI)	*P* value	
Demographic characteristics							
Sex							
Female	1 [Reference]	NA	1 [Reference]	NA	1 [Reference]	NA	NA
Male	1.11 (1.07-1.16)	<.001	1.17 (1.12-1.23)	<.001	1.12 (1.01-1.24)	.03	.24
Race and ethnicity							
Asian or Pacific Islander	0.80 (0.67-0.96)	.01	1.07 (0;.89-1.29)	.49	1.10 (0.73-1.66)	.65	NA
Hispanic	0.86 (0.67-1.09)	.22	0.90 (0.66-1.23)	.52	0.79 (0.33-1.91)	.60	
Non-Hispanic Black	0.89 (0.83-0.96)	.001	0.84 (0.77-0.92)	<.001	1.15 (0.98-1.34)	.09	.01
Non-Hispanic White	1 [Reference]	NA	1 [Reference]	NA	1 [Reference]	NA	NA
Obesity	1.16 (1.11-1.21)	<.001	1.10 (1.05-1.16)	<.001	1.22 (1.09-1.35)	<.001	.16
Adenoma onset age, y							
0-49	1.14 (1.07-1.22)	<.001	0.79 (0.73-0.86)	<.001	1.11 (0.98-1.26)	.11	<.001
≥50	1 [Reference]	NA	1 [Reference]	NA	1 [Reference]	NA	NA
Family history of polyps	1.15 (1.03-1.29)	.01	1.45 (1.28-1.65)	<.001	1.06 (0.79-1.42)	.69	.02
Family history of colorectal cancer	1.26 (1.20-1.33)	<.001	1.44 (1.35-1.53)	<.001	1.34 (1.17-1.53)	<.001	.01

Abbreviation: NA, not applicable.

^a^ Adjusted for age at the first polypectomy, years at the first polypectomy (categorized as <2005, 2005-2009, 2010-2014, 2015-2019, or 2020-2024), sex, and race and ethnicity.

### Associations With Adenoma Characteristics

All the associations of adenoma recurrence with adenoma characteristics showed significant heterogeneity across follow-up periods (Table 3). Histopathological features exhibited larger effect sizes than demographic factors with marked temporal heterogeneity. High-grade dysplasia showed the largest early association (aHR, 4.00; 95% CI, 3.56-4.50; *P* < .001) with complete midterm and late attenuation. Villous histology exhibited biphasic patterns: pronounced early risk (aHR, 2.89; 95% CI, 2.63-3.18; *P* < .001), midterm normalization (aHR, 1.07; 95% CI, 0.92-1.23; *P* = .37), and late reemergence (aHR, 2.71; 95% CI, 2.15-3.41; *P* < .001). Tubulovillous histology showed elevated early (aHR, 2.78; 95% CI, 2.54-3.04; *P* < .001) with subsequent protective midterm (aHR, 0.74; 95% CI, 0.61-0.88; *P* = .001) association. Serrated and mixed histology both increased early risk (aHR, 1.32; 95% CI, 1.20-1.46 and aHR, 1.53; 95% CI, 1.45-1.61, respectively; both *P* < .001), with mixed subtypes subsequently showing midterm protection (aHR, 0.86; 95% CI, 0.80-0.92; *P* < .001).

**Table 3.** Adjusted Hazard Ratios (HR) for Adenoma Recurrence by Adenoma Characteristics Across Time Periods^a^

Variables	<5 Years		5-10 Years		>10 Years		*P* value for Heterogeneity
	HR (95% CI)	*P* value	HR (95% CI)	*P* value	HR (95% CI)	*P* value	
Adenoma type							
Tubular	1 [Reference]	NA	1 [Reference]	NA	1 [Reference]	NA	NA
Villous	2.89 (2.63-3.18)	<.001	1.07 (0.92-1.23)	.37	2.71 (2.15-3.41)	<.001	<.001
Tubulovillous	2.78 (2.54-3.04)	<.001	0.74 (0.61-0.88)	.001	1.03 (0.73-1.45)	.86	
Serrated	01.32 (1.20-1.46)	<.001	0.89 (0.78-1.02)	.09	1.05 (0.77-1.42)	.77	
Not defined	0.60 (0.57-0.64)	<.001	0.68 (0.64-0.73)	<.001	0.87 (0.76-0.99)	.04	
Mixed	1.53 (1.45-1.61)	<.001	0.86 (0.80-0.92)	<.001	1.04 (0.90-1.21)	.60	
Dysplasia							
Low grade	1 [Reference]	NA	1 [Reference]	NA	1 [Reference]	NA	NA
High grade	4.00 (3.56-4.50)	<.001	0.99 (0.71-1.39)	.96	1.04 (0.50-2.17)	.91	<.001
No. of polyps							
<3	1 [Reference]	NA	1 [Reference]	NA	1 [Reference]	NA	NA
≥3	1.95 (1.85-2.06)	<.001	1.18 (1.08-1.29)	<.001	1.18 (0.95-1.47)	.14	<.001
Not defined	0.64 (0.61-0.68)	<.001	0.90 (0.86-0.95)	<.001	0.88 (0.79-0.99)	.04	
Adenoma size, mm							
<10	1 [Reference]	NA	1 [Reference]	NA	1 [Reference]	NA	NA
≥10	1.90 (1.79-2.01)	<.001	0.95 (0.86-1.06)	.37	1.07 (0.86-1.33)	.54	<.001
Not defined	0.66 (0.63-0.69)	<.001	0.86 (0.82-0.91)	<.001	0.88 (0.79-0.99)	.03	
Adenoma type							
Low risk	1 [Reference]	NA	1 [Reference]	NA	1 [Reference]	NA	NA
High risk	2.72 (2.61-2.84)	<.001	1.16 (1.09-1.24)	<.001	1.51 (1.33-1.73)	<.001	<.001

Abbreviation: NA, not applicable.

^a^ Adjusted for age at the first polypectomy, years at the first polypectomy (categorized as <2005, 2005-2009, 2010-2014, 2015-2019, or 2020-2024), sex, race and ethnicity, obesity, use of nonsteroidal anti-inflammatory drugs, use of aspirin, family history of colorectal cancer, and family history of polyps.

Multiple adenomas (≥3) demonstrated stronger early risk (aHR, 1.95; 95% CI, 1.85-2.06) with attenuated but persistent midterm elevation (aHR, 1.18; 95% CI, 1.08-1.29; *P* < .001) and nonsignificant late outcomes. Adenomas 10 mm or more showed elevated early risk (aHR, 1.90; 95% CI, 1.79-2.01; *P* < .001) with complete attenuation thereafter. High-risk adenomas exhibited marked early association (aHR, 2.72; 95% CI, 2.61-2.84; *P* < .001) with persistent midterm (aHR, 1.16; 95% CI, 1.09-1.24; *P* < .001) and late elevation (aHR, 1.51; 95% CI, 1.33-1.73; *P* < .001).

### Stratified Analyses by Sex and Ethnicity

Further analyses of the association of adenoma recurrence with adenoma characteristics were stratified by sex and ethnicity. A significant heterogeneity of the association with adenoma type was observed between men and women (Table 4) (*P *for heterogeneity < .001). Female patients exhibited stronger associations during early (<5 years: aHR, 2.86; 95% CI, 2.68-3.04; vs 2.61; 95% CI, 2.46-2.76) and late (>10 years) surveillance (aHR, 1.73; 95% CI, 1.43-2.08; *P* < .001 vs male patients: 1.29; 95% CI, 1.06-1.58; *P* = .01). No other significant heterogeneity by sex and ethnicity were found.

**Table 4.** Adjusted Hazard Ratios (HR) for High-Risk Adenoma Type Across Time Periods by Sex^a^

Variable	<5 y		5-10 y		>10 y		*P* value for heterogeneity
	HR (95% CI)	*P* value	HR (95% CI)	*P* value	HR (95% CI)	*P* value	
Men	2.61 (2.46-2.76)	<.001	1.18 (1.07-1.29)	.001	1.29 (1.06-1.58)	.01	NA
Women	2.86 (2.68-3.04)	<.001	1.15 (1.04-1.27)	.01	1.73 (1.43-2.08)	<.001	<.001

Abbreviation: NA, not applicable.

^a^ Adjusted for age at the first polypectomy, years at the first polypectomy (categorized as <2005, 2005-2009, 2010-2014, 2015-2019, 2020-2024), race and ethnicity, obesity, use of nonsteroidal anti-inflammatory drugs, use of aspirin, family history of colorectal cancer, and family history of polyps.

## Discussion

The current Vanderbilt Polypectomy Cohort of 59 667 adults who underwent colonoscopic polypectomy suggests that colorectal adenoma recurrence is demographically patterned and temporally heterogeneous, rather than constant over time. Current guidelines prioritize polyp characteristics while excluding demographic factors; our findings demonstrate that demographic variables and histopathologic features are jointly associated with recurrence risk, with neither independently sufficient for optimal stratification. Early recurrence (<5 years) was associated with male sex, obesity, early-onset adenoma, and high-grade dysplasia, whereas midterm risk was dominated by family history, and late-phase risk (>10 years) was characterized by the resurgence of villous histology and a specific, marked elevation in recurrence among female patients with high-risk adenomas. These findings show that postpolypectomy surveillance should incorporate time-dependent and population-specific risk rather than applying uniform intervals to all patients.

Male patients exhibited sustained recurrence risk across all intervals, consistent with studies linking male sex to advanced neoplasia.^21,22,23,24^ Demographic effect sizes, though modest, revealed clinically meaningful heterogeneity in recurrence timing overlooked by current guidelines. Crucially, our sex-stratified analysis revealed that while both sexes showed attenuated midterm risk (women: aHR, 1.15; 95% CI, 1.04-1.27; men: aHR, 1.18; 95% CI, 1.07-1.29), women with high-risk adenomas exhibited a marked late-phase resurgence significantly exceeding that of men (>10 years: aHR, 1.73; 95% CI, 1.43-2.08 vs 1.29; 95% CI, 1.06-1.58). This female-specific late-term elevation contributed to significant overall heterogeneity (*P* < .001). Consequently, this challenges USMSTF’s uniform surveillance cessation policies,^9^ suggesting that high-risk female patients may benefit from extended surveillance duration beyond current recommendations to capture this late-phase latency.

Non-Hispanic Black patients exhibited sustained lower recurrence during early and midterm surveillance (aHR, 0.89; 95% CI, 0.83-0.96 and 0.84; 95% CI, 0.77-0.92, respectively), with nonsignificant late-phase attenuation (aHR, 1.15; 95% CI, 0.98-1.34; *P* = .09). This lower observed recurrence likely reflects structural disparities—specifically lower adenoma detection rates during surveillance or loss to follow-up—rather than biological protection.^8,25,26^ Current guidelines inadequately address this heterogeneity, with ESGE omitting surveillance for villous adenomas less than 10 mm.^2,13,14,36^ Rather than relaxed surveillance, non-Hispanic Black patients may require quality-adjusted strategies ensuring high-ADR endoscopists. Structural inequities, including unequal health care access,^27,28,29^ necessitate standardized care and prospective studies.

Early-onset adenomas, more frequent in Hispanic and Asian and Pacific Islander, correlated with excess early recurrence (aHR, 1.14; 95% CI, 1.07-1.22), consistent with rising early-onset neoplasia,^28,30^ but this risk normalized after 5 years. Despite USMSTF’s 2022 recommendation for screening at age 45 years,^31,32^ guidelines do not account for age at adenoma detection as a recurrence modifier. Family history of colorectal cancer conferred a clinically meaningful, persistent elevation peaking in midterm recurrence (aHR, 1.44; 95% CI, 1.35-1.53). In contrast, family history of polyps had minimal immediate association but surged at 5 to 10 years (aHR, 1.45; 95% CI, 1.28-1.65), supporting sustained surveillance and indicating temporally distinct trajectories.^33,34,35,36,37^ Notably, obesity was persistently associated with recurrence across all intervals (>10 years: aHR, 1.22; 95% CI, 1.09-1.35). This challenges assumptions of metabolic risk attenuation and suggests a cumulative, long-term oncogenic pressure that does not dissipate over time.^6,38,39^ Together, these patterns show familial and metabolic risk factors operate on distinct temporal scales and should inform tailored colonoscopy intervals for less than 5-year and 5- to 10-year surveillance windows.

Effect size magnitudes revealed that histopathologic features were the dominant recurrence variables. High-grade dysplasia exhibited the single largest effect size (aHR, 4.00; 95% CI, 3.56-4.50) but was strictly limited to early recurrence (<5 years), whereas villous histology demonstrated a biphasic risk pattern: high early risk (aHR, 2.89; 95% CI, 2.63-3.18), normalization at midterm, and a significant resurgence in late risk (aHR, 2.71; 95% CI, 2.15-3.41), surpassing adenoma number, size, and demographic factors. The distinct temporal profiles of high-grade dysplasia (early hazard) vs villous histology (biphasic or late hazard) challenge the ESGE’s simplified grouping of “advanced” adenomas. USMSTF flags villous histology as high-risk,^9,40^ but ESGE’s omission of villous features^2^ may underestimate long-term risk. The late-phase resurgence of villous risk underscores the need for prolonged follow-up for these specific lesions.

Adenoma recurrence risk is heterogeneous and dynamic, inadequately captured by static guideline frameworks. Time-varying coefficient Cox models revealed evolving risk trajectories; obesity exerted sustained pressure, early-onset lesions shifted from excess to deficit risk, and non-Hispanic Black patients showed patterns consistent with detection disparities. High-risk adenomas showed a late-phase resurgence (>10 years), particularly in women. Villous morphology maintained a latent hazard that reemerged after a decade, challenging ESGE’s exclusion of villous features less than 10 mm.^2^ A recent cohort noted higher adenoma burden in non-Hispanic Black patients at older ages, suggesting biology-care interactions,^41^ while a study found no race effect with uniform colonoscopy quality.^29^ These findings reconcile discordant results, emphasizing dynamic, race-specific hazards. The 2020 USMSTF lengthened surveillance for low-risk adenomas but retained uniform high-risk intervals, and ESGE’s simplified high-risk definitions^2^ overlook sustained villous risk. Integrating temporal coefficients and heterogeneity metrics could align surveillance with evolving patient risk, enhancing precision and equity in CRC prevention.

### Limitations

While this study’s cohort of 59 667 patients, followed up for as long as 25 years, robustly examines colorectal adenoma recurrence across diverse subgroups using validated natural language processing for histologic and clinical feature extraction, enabling stratified analyses by sex, race and ethnicity, onset age, and adenoma subtype, this single-center study with predominantly non-Hispanic White patients may lack generalizability, and its retrospective design risks selection bias and unmeasured confounding (eg, diet, smoking, socioeconomic status, health care access, and colonoscopy quality). Socioeconomic status and lifestyle exposures (including tobacco, alcohol, physical activity, and diet) were not consistently available in EHRs, precluding their inclusion. Residual confounding from these factors may partially explain observed racial, sex-specific, and age-related heterogeneity in recurrence risk. Modest demographic effect sizes may reflect residual confounding from unmeasured socioeconomic and lifestyle factors. Additionally, variable surveillance frequency may affect recurrence detection, and reliance on VUMC network colonoscopies risks outcome misclassification if interval colonoscopies occurred externally.

## Conclusions

In this cohort study of 59 667 adults, colorectal adenoma recurrence demonstrated distinct temporal heterogeneity rather than constant risk. While high-grade dysplasia was a dominant early association, villous histology exhibited a specific late-phase resurgence (>10 years) that static guidelines may overlook. Obesity conferred sustained risk across all intervals, challenging assumptions of diminishing metabolic influence. Most notably, female patients with high-risk adenomas experienced a significant late-term recurrence surge that exceeded that of male patients, suggesting current surveillance cessation policies may underserve women. These findings support a paradigm shift toward dynamic, time-dependent surveillance that extends monitoring for patients with specific histologic and demographic risk profiles.

## Notes

Supplement 1.
eMethods.

Supplement 2.
Data Sharing Statement
