# Supplementary material for: Demographic and Clinicopathologic Factors Associated With Colorectal Adenoma Recurrence
Source: JAMA Netw Open. 2026 Feb 4;9(2):e2556853. doi: 10.1001/jamanetworkopen.2025.56853 (PMC12873766; doi:10.1001/jamanetworkopen.2025.56853)
Supplement: Supplement 1. — eMethods. [file jamanetwopen-e2556853-s001.pdf]

Supplemental Online Content

Awan UA, Song Q, Ciombor KK, et al. Demographic and clinicopathologic factors associated with colorectal adenoma recurrence. *JAMA Netw Open*. 2026;9(2):e2556853. doi:10.1001/jamanetworkopen.2025.56853

eMethods.

This supplemental material has been provided by the authors to give readers additional information about their work.

© 2026 Awan UA et al. *JAMA Network Open*.

eMethods.

Data Extraction and Variable Definitions

Variables were extracted and categorized as follows: Demographic factors included age (stratified as ≤50, 51–75, ≥76 years), sex (male/female), self-reported race/ethnicity (non-Hispanic White [NHW], NHB, Hispanic, Asian/Pacific Islander [API]), family history of polyp, and family history of CRC. Clinical variables included obesity (BMI ≥30 kg/m^2, defined via ICD-9/ICD-10 codes), medication use (e.g., aspirin, NSAIDs) and screening colonoscopies (procedures without biopsy). Adenoma characteristics encompassed histological subtype (tubular, tubulovillous, villous, serrated, mixed), number (<3 vs. ≥3), size (<10 mm vs. ≥10 mm) and dysplasia grade (low/high). Mixed adenoma was defined as the presence of more than one histologic polyp subtype (e.g., tubular, villous, serrated) within a single patient at the time of index colonoscopy. For analysis of polyp onset age, patients were categorized into two groups: early onset (0–49 years), and late onset (≥50 years).

Furthermore, a secure LLM managed by VUMC was used to extract polyp characteristics—including type, number, and size—from unstructured histopathology reports and clinical notes. The LLM utilized case-insensitive keyword searches to identify relevant terms such as *polypectomy*, *adenoma*, *tubul*, *villous*, *sessile*, *plastic*, and *serrated* within clinical documentation (Supplement 1). Variables with incomplete or unextractable data (e.g., missing histology, size, or multiplicity) were systematically labeled as *Not Defined* to preserve data integrity and transparency.

Ethical Considerations

This study was reviewed and exempted from human subjects' research by the Institutional Review Board at Vanderbilt University Medical Center (IRB #250158). Patient confidentiality was maintained through data de-identification, and all procedures adhered to relevant ethical standards. Data was stored and analyzed in a secure, encrypted, access-controlled database.

Pathology Report Extraction

© 2026 Awan UA et al. *JAMA Network Open*.

This protocol outlines the methodology for extracting polyp characteristics from pathology reports to characterize polyp type, number, size, and dysplasia for analysis in the Polypectomy Cohort study. Pathology reports were identified using specific keywords to ensure relevance, while unrelated notes were excluded to minimize noise. Extracted variables were structured in JSON format for consistency and downstream analysis.

Keywords Used for Identification:

- polypectom
- adenoma
- tubul
- villous
- sessile
- plastic
- serrated

Exclusion Criteria (to Filter Unrelated Notes):

- Discharge
- Re:
- Anesthesia
- Appointment
- PROBLEM LIST
- Endoscopy Nurse Note
- Consent - Surgery/Procedure
- Patient Instructions

Extracted Variables:

1. **Adenoma Type & Histological Type:** Categorized as:
    - Adenomatous: Tubular, Villous, Tubulovillous
    - Serrated Polyps: Hyperplastic, Sessile serrated, Traditional serrated, Unclassified serrated adenomas
    - Non-neoplastic: Inflammatory, Hamartomatous
    - Mixed Type: Multiple polyp types
2. **Number of Polyps:** Multiple fragments in one position or specimen counted as 1 polyp.
3. **Maximum Size of Largest Adenoma or Fragment:** Measured in millimeters (mm).

© 2026 Awan UA et al. *JAMA Network Open*.

4. **Dysplasia Type:** Classified as Low-grade, High-grade, or "Not specified" if unspecified.

Output the result in Json format without explanation
Two Examples:

{
"histological_type" : "Adenomatous: Villous",
"number_of_polyp" : 1,
"max_size_of_polyp" : "12 mm",
"dysplasia_type" : "High-grade"
}

{
"histological_type" : "Mixed Type: Tubular, Sessile serrated",
"number_of_polyp" : 3,
"max_size_of_polyp" : "10 mm",
"dysplasia_type" : "Low-grade"
}"

© 2026 Awan UA et al. *JAMA Network Open*.
