# Supplementary material for: Demographic and Clinicopathologic Factors Associated With Colorectal Adenoma Recurrence
Source: JAMA Netw Open. 2026 Feb 4;9(2):e2556853. doi: 10.1001/jamanetworkopen.2025.56853 (PMC12873766; doi:10.1001/jamanetworkopen.2025.56853)
Supplement: Supplement 2. — Data Sharing Statement [file jamanetwopen-e2556853-s002.pdf]

Data Sharing Statement

Awan. Demographic and Clinicopathologic Factors Associated with Colorectal Adenoma Recurrence. *JAMA Netw Open*. Published February 02, 2026.
doi:10.1001/jamanetworkopen.2025.56853

Data
Data available: Yes
Data types: Data (not involving human participants)
How to access data: https://github.com/XingyiGuo/CRA_recurrence
When available: With publication

Supporting Documents
Document types: None

Additional Information
Who can access the data: anyone requesting the data
Types of analyses: for any purpose
Mechanisms of data availability: with investigator support
